# Tumor necrosis associates with aggressive breast cancer features, increased hypoxia signaling and reduced patient survival

**DOI:** 10.1038/s41598-025-29905-3

**Published:** 2025-11-27

**Authors:** Astrid A. Syrtveit, Lise M. Ingebriktsen, Amalie F. Tegnander, Lars A. Akslen, Elisabeth Wik, Erling A. Hoivik

**Affiliations:** 1 Centre for Cancer Biomarkers CCBIO, Department of Clinical Medicine, Section for Pathology, University of Bergen, Bergen, Norway; 2 Department of Pathology, Haukeland University Hospital, Bergen, Norway

**Keywords:** Breast Cancer, Necrosis, Signature Score, Proliferation, Stemness, Hypoxia, Cancer, Oncology, Pathogenesis

## Abstract

In the growing tumor, hypoxia may lead to tumor necrosis which is associated with more aggressive tumor features and reduced patient survival. However, there are shortcomings in our understanding of biological features and clinical significance linked to tumor necrosis. We therefore analyzed transcriptome mRNA expression profiles from primary breast tumors from TCGA and METABRIC (n = 2289) including TCGA mutational data (n = 409). We employed bioinformatics analysis and independent literature-based signatures for validation to identify alterations unique to necrosis. Necrosis was associated with aggressive tumor features and strongly predicted the basal-like breast cancer phenotype. Increased tumor cell proliferation, hypoxia, stemness, and epithelial-to-mesenchymal transition (EMT) were observed in tumors with necrosis. From gene expression data, we constructed a novel Breast Cancer Necrosis Signature (BCNS) score that was a strong predictor of the basal-like phenotype but also conveyed information relevant to the luminal subtypes of breast cancer, including prognosis. Mutational profiling pointed to enrichment of *TP53* and *PIK3CA* mutations in tumors with and without necrosis, respectively. This study confirms the association of breast cancer necrosis with aggressive tumor features and reduced survival, and points to the BCNS score as a potential biomarker that should be further explored and validated to improve breast cancer diagnosis and management.

**Supplementary Information:**

The online version contains supplementary material available at 10.1038/s41598-025-29905-3.

## Introduction

The complex interactions between tumor cells and the surrounding tumor microenvironment contribute to cancer progression^1^. Tumor cells that reside in the inner tumor mass suffer from microenvironment shortcomings, such as nutrient deficiency and hypoxia^2–5^. In pace of the growing tumor, the need for nutrients and oxygen increases, but in case of insufficient blood supply, metabolic and hypoxic stress may lead to cell death, a process described as tumor necrosis^6^. Necrosis is a nonregulated cell death process with no specific molecule involved in the process, contrasting regulated (programmed) necrotic cell death processes of necroptosis, pyroptosis and ferroptosis, caused by specific proteins^7,8^. Tumor necrosis is an indicator of aggressive tumor features in several cancers^9–11^, including breast cancer^12–16^, where tumor necrosis is associated with large tumor size, presence of positive lymph node metastases and poor outcome^6,9,17,18^. However, conflicting findings of tumor necrosis has been reported within breast cancer, ranging from positive correlations between necrosis and tumor aggressiveness^19,20^, to studies showing no direct association between tumor necrosis and prognosis^13,21^. These variant observations may be due to molecular subtype differences. In the triple-negative subtype of breast cancer, studies have reported incidences of tumor necrosis in the range 45–56%^22,23^, potentially explained by high tumor cell proliferation observed in this subtype^24,25^.

Although the histopathological evaluation of necrosis in breast cancer lesions may provide prognostic information useful for diagnosis and patient management, there is limited information on the biological underpinnings of this process. In this study, we investigate molecular, genetic, and clinico-pathological variables related to breast cancer necrosis, among others, through a necrosis-derived mRNA expression signature in the TCGA and METABRIC cohorts. Our results demonstrate a clear link between necrosis and aggressive breast cancer features, poor survival and oncogenic pathway signaling.

## Material and methods

### Data sets and patient cohorts

We employed three public datasets of mRNA gene expression from primary tumors spanning a total of 2289 breast cancer patients, including clinical information: First, The Cancer Genome Atlas (TCGA) Invasive Breast Cancer data (TCGA BRCA, n = 520)^26^ and next, the Molecular Taxonomy of Breast Cancer International Consortium (METABRIC) independent discovery and validation cohorts (full datasets of n = 997 and n = 995, respectively)^27^. Additionally, for the TCGA BRCA cohort, information on tumor necrosis and mutational status by Illumina whole exome sequencing data were available^26,28^. Information on the TCGA HER2 status was available as “*her2_status_final*” variable evaluated as “*Positive*” if HER2 overexpression/amplification, and “*Negative*” if no overexpression/amplification from IHC assessment^27^. In the study by Thennavan *et al.*, updated and complete histology and morphologic features were presented for TCGA-BRCA samples with consensus by the International Breast Cancer Pathology Expert Committee^28,29^. There, the morphological necrosis feature was graded by at least two pathologists to “*Present*” or “*Absent*” by assessing images from the TCGA digital slide archive (http://cancer.digitalslidearchive.net). Intrinsic molecular subtypes, deduced from the PAM50 classification algorithm were available for all cohorts^30^. The TCGA BRCA datasets were filtered to contain only female patients. The normal-like molecular subtype was excluded in all analyses. Hence, the final female breast cancer dataset without normal-like subtypes of the TCGA BRCA and METABRIC discovery/validation cohorts were n = 483, and n = 939/845 patients, respectively.

### Gene expression analyses

#### Differential gene expression and gene set enrichment analysis

All gene expression data was log2 transformed. In case of multiple probes in the microarray mRNA expression datasets covering the same gene, we collapsed these according to the max-probe approach^31^. To identify genes differentially expressed between samples with morphologic necrosis present or absent, we applied the method of Significance Analysis of Microarrays (SAM)^29^. Gene set enrichment analyses were performed by employing the Gene Set Enrichment Analysis tool (GSEA; www.broadinstitute.org/gsea)^31^ with gene set modules from the Molecular Signatures Database (MSigDB, v2022.1 human). Investigations in MSigDB included classes of Curated gene sets (C2), Gene Ontology gene sets (C5), Oncogenic Signature gene sets (C6), and Hallmark gene sets (H). Enrichment analysis was performed with the gene-list comprising the BCNS as described below (Over-representation analysis; ORA), using the oncoEnrichR tool with the functional enrichment module^32^.

#### Gene expression signatures

A gene expression signature reflecting the expression pattern of tumor necrosis was made from the genes differentially expressed between tumors with and without tumor necrosis, with cut-offs of fold change ≥|2.5| and FDR < 0.008%. By subtracting the sum of gene expression values of genes with lower expression (“*DOWN*”) from the sum of genes showing higher expression values (“*UP*”) in tumors with necrosis, a Breast Cancer Necrosis Signature (BCNS) mRNA score was calculated for each case. The calculation of the score (26 genes with higher expression and 60 genes with lower gene expression) is described as:

BCNS score = Σ(Genes with elevated expression (UP))—Σ(Genes with reduced expression (DOWN)).

The BCNS score was dichotomized into “high”/ “low” based on positive/negative score values (cut-off at zero) by visual inspection of the plotted data.

Additional signatures reflecting tumor cell proliferation were calculated, including a Stathmin^33^, Oncotype DX^34^, and a proliferating cell nuclear antigen (PCNA) score^35^. We also included signatures reflecting hypoxia^36–43^, a Nestin score reflecting stemness^44^, an EMT score^45^, a luminal progenitor score, a mature luminal score, and a mammary stem cell score^46^. The TCGA-HER2-index score was available from^47^. Signature scores were calculated as described in their original publications, or else by a summarizing score of the genes in the signature^33^. Unsupervised hierarchical clustering with complete linkage and Euclidean distance was done for visualization of the signature scores.

### Mutational analysis

From the TCGA BRCA dataset, 409 unique cases also had annotated mutational data available, overlapping with the gene expression data, after filtering off duplicated samples. We analyzed mutational data using R software (v.4.3.0; Vienna, Austria) using the maftools package^48^ to compare mutation profiles by tumor necrosis status.

### Statistical methods

Data were analyzed using the SPSS Statistics for Windows, Version 27.0 (IBM Corp., Armonk, NY, USA). Statistical significance was assessed at the two-sided 5% level. Associations between categorical variables were evaluated using the Pearson’s χ^2^ test. Non-parametric correlations were tested by Spearman’s rank coefficient. Continuous variables were compared between two groups using the Mann–Whitney U test, and between more than two groups using Kruskal–Wallis H test. Univariate survival analyses were carried out using the Kaplan–Meier method with significance determined by the log-rank test. Entry date was set to the date of diagnosis. Breast cancer disease specific survival was employed as endpoint in survival analyses. Patients who died from other causes than cancer were censored at the date of death. Multivariate survival analyses were performed on both continuous and categorized Breast Cancer Necrosis Signature score in the METABRIC cohorts, using Cox’ proportional hazards regression model. Only patients with information on all variables were included in the analyses.

## Results

### Morphologic tumor necrosis associates with aggressive breast cancer features

**Fig. 1:**
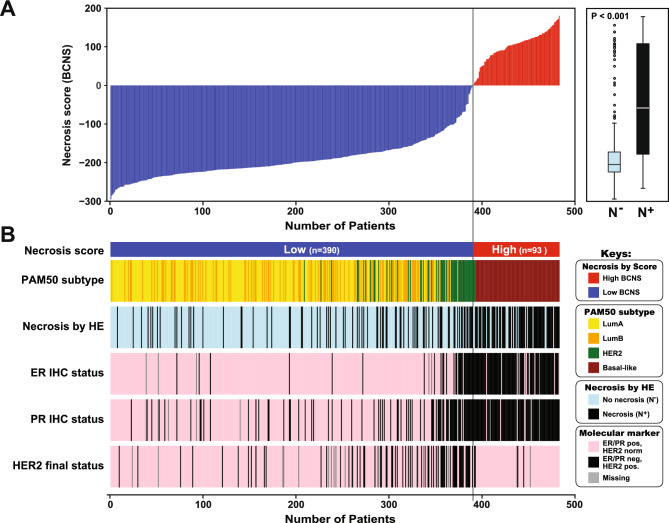
**Morphological tumor necrosis and relation to female breast cancer features and a necrosis-specific gene expression signature score.** (**A**) Left: The Breast Cancer Necrosis Signature (BCNS) score (ordered from low to high) calculated from mRNA expression data. Right: Boxplot of the BCNS score levels assessed by histopathological evaluation (HE) of necrosis status^28^. (**B**) A high breast cancer necrosis score (n = 93) is associated with basal like PAM50 subtype, and loss/low levels (negativity) of Estrogen receptor (ER) alpha and Progesterone receptor (PR), and HER2 positivity, compared to the patient group with low score (n = 390). Data from the TCGA BRCA study^26^, with normal-like molecular subtype excluded from analysis, n = 483.

This study leverages on previous morphologic evaluation of tumor necrosis in primary tumors of the TCGA breast cancer cohort^28^. Here, we aimed to describe molecular tumor features in breast cancer with tumor necrosis. We first examined how tumor necrosis was related to molecular markers and subtypes (PAM50) of breast cancer using the TCGA data and found the presence of morphologic tumor necrosis to be associated with molecular features related to more aggressive tumor phenotypes, like estrogen receptor (ER [alpha]) and progesterone receptor (PR) negativity and the Basal-like molecular subtype (all P < 0.001, Table 1, and Fig. 1).

**Table 1:** **The relation between morphologic (HE) necrosis and clinico-pathologic features in the TCGA cohort (BRCA, n = 483).**

Variable	Necrosis absent (N^-^)	Necrosis present (N^+^)	P-value†
No. patients	n = 331	n = 152	
ER status (IHC)			
Positive	292 (89.6%)	73 (48.7%)	< 0.001
Negative	34 (10.4%)	77 (51.3%)	
PR status (IHC)			
Positive	255 (78.7%)	57 (37.7%)	< 0.001
Negative	69 (21.3%)	94 (62.3%)	
HER2 (IHC)			
Negative	277 (86.3%)	118 (81.9%)	0.262
Positive	44 (13.7)	26 (18.1)	
Molecular subtype (PAM50)			
Luminal A	186 (56.2%)	33 (21.7%)	< 0.001
Luminal B	90 (27.2%)	26 (17.1%)	
HER2-enriched	32 (9.7%)	24 (15.8%)	
Basal-like	23 (6.9%)	69 (45.4%)	

^†^Chi-square test, two sided. n = number of patients. Missing cases: ER, n = 7; PR, n = 8; HER2, n = 18.

### A novel Breast Cancer Necrosis Signature (BCNS) score associates with aggressive cancer features and clinical outcome

To corroborate our findings of aggressive molecular phenotypes in tumors with necrosis, we derived a Breast Cancer Necrosis Signature (BCNS) score from genes differentially expressed between patient groups with and without tumor necrosis (morphologic assessment), using the TCGA BRCA cohort with microarray mRNA expression data. We constructed this signature from 60 genes with elevated expression and 26 genes with reduced gene expression from our SAM-analysis using fold change ≥|2.5| and FDR < 0.008% (top-ranked genes in Table 2; full list in Supplementary Data) and considered this signature to be a surrogate marker for morphologic (HE) tumor necrosis assessment. The necrosis signature (BCNS) score demonstrated significantly higher scores in tumors with morphologic necrosis present (N^+^) compared to tumors without necrosis (P < 0.001, Fig. 1A, right; n = 483). Tumor necrosis was linked to aggressive tumor features, including loss of ER- , PR expression, HER2 positivity and appeared almost exclusively associated (98%) with the Basal-like molecular breast cancer subtype (Fig. 1B and Table 3). By our approach, BCNS-low cases overlap with 21.0% of cases that were morphologically N^+^ (82/390), while for the BCNS-high 75.2% cases were N^+^ (70/93) demonstrating a strong significant association between the two variables (p < 0.001; Supplementary Table S1). Using the oncoEnrichR tool^32^, we find the genes comprising the 86-gene BCNS signature to be highly relevant, as the Gene Ontology “Molecular Function” from MSigDB^31^ are dominated by breast cancer terms with descriptions connected to estrogen-related signaling (Supplementary Figure S1A).

**Table 2:** **Ten most upregulated- and downregulated genes by necrosis groups.**

Gene name	Gene symbol	Functions	Fold change
Genes differentially expressed, up†			
Ecto-ADP-ribosyltransferase 3	ART3	Increased cell proliferation, invasion and sustained survival of cancer cells	3.6
Phosphoerine aminotranferase 1	PSAT1	Increased cell proliferation	3.6
Rhophilin-associated tail protein	ROPN1	Cancer testis antigen. Migration, invasion and metastasis	3.4
SRY-Box transcription factor 11	SOX11	Cancer testis antigen. Reactivation of embryonic signals. Epithelial-to- mesenchymal transition, migration and invasion	3.3
Matrix metallopeptidase 12	MMP12	Tumor angiogenesis, migration, invasion and regulation of immune surveillance	3.2
Protein phosphatase 1 regulatory inhibitor subunit 14C	PPP1R1 4C	Protein synthesis, metabolism, cell proliferation	3.2
Rhophilin associated tail protein 1B	ROPN1B	Cancer testis antigen	3.1
Horma Domain Containing protein 1	HORMA D1	Cancer testis antigen	3.0
Chemokine C-X-C ligand 1	CXCL1	Chemoattractant for neutrophils	2.9
LEM Domain Containing protein 1	LEMD1	Cancer testis antigen Proliferation, invasion, migration	2.8
Genes differentially expressed, down†			
Anterior gradient 3, protein disuphide isomerase family member	AGR3	Tumor suppressor	– 10.9
Steroid receptor associated and regulated protein	C1orf64 /SRARP	Tumor suppressor	– 6.2
Estrogen receptor 1	ESR1	Encodes the estrogen receptor	– 5.9
Signal peptide CUB domain and EGF like domain containing 2	SCUBE2	Suppresses breast tumor cell proliferation	– 5.0
Forkhead Box 1A	FOXA1	Metabolism and cell differentiation	– 5.0
Anterior gradient 2	AGR2	Cell migration, cellular transformation, metastasis. Is also a p53 inhibitor	– 4.6
N-acetyltransferase	NAT1	Affects cancer cell proliferation and survival. Regulates HIF1-α	– 4.3
GDNF family receptor alpha 1	GFRA1	Neuron survival and differentiation	– 4.3
secretoglobin family 2A member 2	SCGB2A 2	Overexpression decreases migration and invasion	– 3.9
(Tre-2/Bub2/Cdc16) domain family	TBC1D9	Cellular proliferation, mitosis, migration, extracellular matrix remodeling and membrane repair	– 3.9

^†^Direction of expression change in N^+^ (relative to N^-^).

Selected genes most differentially expressed (by fold change) in tumors with and without tumor necrosis (SAM analysis). Top ten genes with the highest elevated expression (denoted UP) or reduced expression (denoted DOWN) in relation to necrosis status. (Data from TCGA, n = 483).

**Table 3:** **Relation between high/low breast cancer necrosis signature (BCNS) score and clinico-pathologic features (TCGA cohort, n = 483)**.

Breast Cancer Necrosis Signature (BCNS) score			
Variable	High (n = 93)	Low (n = 390)	
	n(%)	n(%)	P-value†
ER status (IHC)			
Positive	10 (10.9%)	355 (92.2%)	< 0.001
Negative	81 (88.0%)	30 (7.8%)	
PR status (IHC)			
Positive	5 (5.6%)	307 (79.7%)	< 0.001
Negative	85 (94.4%)	78 (20.3%)	
HER2 (IHC)			
Negative	87 (95.6%)	308 (82.4%)	0.002
Positive	4 (4.4%)	66 (17.6%)	
Molecular subtypes (PAM50)			
Luminal A	0 (0%)	219 (56.2%)	< 0.001
Luminal B	0 (0%)	116 (29.7%)	
HER2-enriched	2 (2.2%)	54 (13.8%)	
Basal like	91 (97.8%)	1 (0.3%)	

^†^Chi square test, two-sided. Missing cases: ER, n = 7; PR, n = 8, HER2, n = 18.

We investigated the Luminal B subtype specifically, where all cases are of low BCNS score, for its relation to the panel of cancer related signature scores. We found that even within this subtype, the BCNS score captured differences in expression of the signatures, although not driven by the major key variables ER, PR and HER2. Interestingly, the two clusters divided by mean showed significantly more often presence of morphological necrosis in the *Luminal_B_BCNS_high* group (P = 0.016) (Supplementary Figure S2A).

To further validate the association of breast cancer necrosis with aggressive tumor features, we assessed the BCNS score in two independent cohorts, the METABRIC discovery and validation cohorts, confirming that high BCNS scores associated with high histologic grade, ER negativity, lymph node metastases, and the basal-like phenotype (Supplementary Table S2). In the METABRIC validation cohort, the associations between the BCNS score and aggressive tumor features were confirmed, except for the association with lymph node metastases, but with the additional observation that the BCNS score associated with large tumor size (P = 0.02, comparing quartile groups; Supplementary Table S2).

**Fig. 2:**
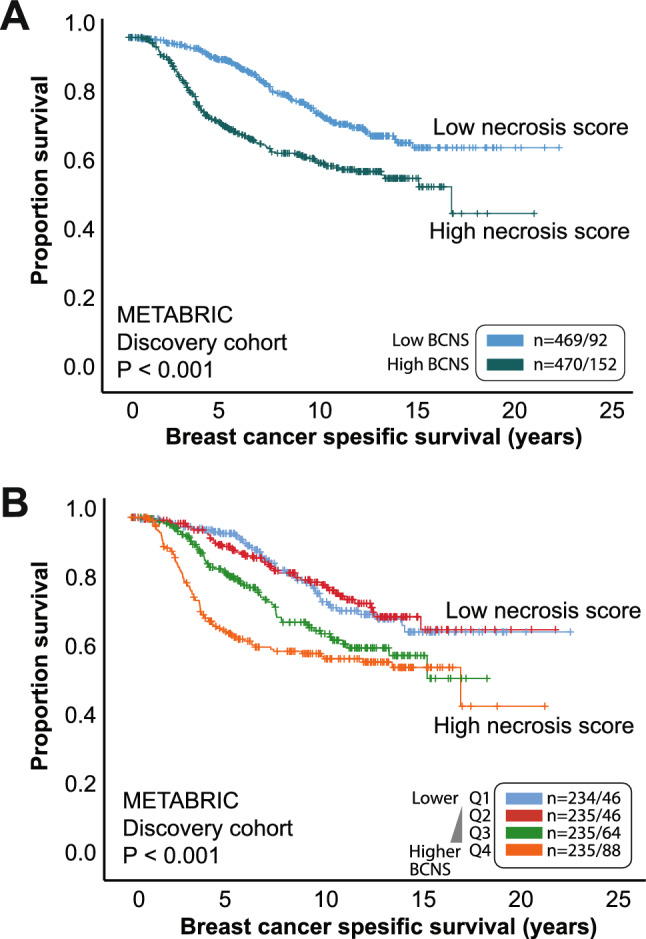
**The Breast Cancer Necrosis Signature (BCNS) score in relation to survival. **A high necrosis score is associated with shorter breast cancer disease-specific survival in the METABRIC cohort evaluated as low–high (**A**) and by quartiles (Q1-4) (**B**). Data from METABRIC discovery cohort, n = 939^27^, with number of cases and events presented in Kaplan–Meier survival curves.

To evaluate the clinical relevance of our tumor necrosis score, we next assessed how the BCNS score related to survival. Because TCGA BRCA microarray data has limited follow-up information, we sought to investigate this in METABRIC data. A high necrosis signature score (BCNS) was significantly associated with poor survival both when dichotomized by mean value or by quartile groups, in both METABRIC discovery and validation datasets (all P < 0.001, Fig. 2 and Supplementary Figure S3). In multivariate analyses, when adjusting for the standard clinico-pathologic variables of tumor size, histologic grade, and lymph node status, we demonstrated independent prognostic impact for the BCNS score in the METABRIC cohorts, both when assessed as continuous- and categorized variables (Table 4 and Supplementary Tables S3-4). Of note, the BCNS score was an independent predictor of survival when adjusted for tumor size, histologic grade, and lymph node status within the Luminal subset (METABRIC Discovery cohort, Supplementary Table S5), but not when including all cases and adding PAM50 to the model.

**Table 4:** **Cox multivariate survival analysis (METABRIC Discovery cohort).**

Variable	n(%)	HR	95% CI	P-value	HR	95.0% CI	P-value
Unadjusted					Adjusted		
Tumor size				< 0.001			0.003
≤ 20 mm	409 (44)	1.0			1.0		
> 20 mm	530 (56)	2.0	1.5–2.6		1.5	1.2–2.0	
Histologic grade				< 0.001			0.06
Grade 1/2	456 (49)	1.0			1.0		
Grade 3	483 (51)	1.9	1.4–2.4		1.3	0.98–1.8	
Lymph node status				< 0.001			< 0.001
Negative	482 (51)	1.0			1.0		
Positive	457 (49)	2.5	1.9–3.2		2.0	1.5–2.7	
BCNS score	939 (100)	1.005	1.003- 1.006	< 0.001	1.003	1.001- 1.005	0.006

HR = Hazard ratio, CI = Confidence interval, n = number of patients.

The Breast Cancer Necrosis Signature (BCNS) score included as continuous variable (n = 939).

Regarding survival by subtypes, the high BCNS was significant across Luminal subtypes applying groups of score-levels by mean score, all four quartiles, or the upper quartile (Q4), driven by stronger effect of the Luminal B subtype but not Luminal A (Supplementary Figure S4). There was no significance for the HER2 or Basal-like subtypes due to few or lack of cases deemed as *BCNS_low*.

### A high BCNS score is a strong predictor of the basal-like phenotype

To follow up on the strong association between BCNS and the basal-like phenotype, we further drilled down on how the BCNS score predicts the basal-like versus non-basal phenotypes. We found a strong prediction of the basal-like phenotype by the BCNS score (83–100% overlap among BCNS-high and basal-like phenotype; P < 0.001, all cohorts; Supplementary Table S2). In multivariate logistic regression analyses, adjusting for cytokeratin 5 (CK5), P-cadherin and epidermal growth factor receptor (EGFR) (by mRNA data), the BCNS score independently predicted the basal-like phenotype (P ≤ 0.003 for BCNS in all cohorts, Supplementary Table S6).

### The BCNS score relates to differences in transcriptome signatures of multiple signaling pathways

We next aimed to explore biological processes accompanying tumor necrosis. We employed Gene Set Enrichment Analyses (GSEA) comparing gene expression patterns of breast cancers with and without necrosis (TCGA BRCA dataset; Supplementary Table S7). Gene sets reflecting proliferation, the processes of plasticity, epithelial-mesenchymal transition (EMT) and features of stemness were enriched in tumors with necrosis, together with hypoxia (Supplementary Table S7).

**Fig. 3:**
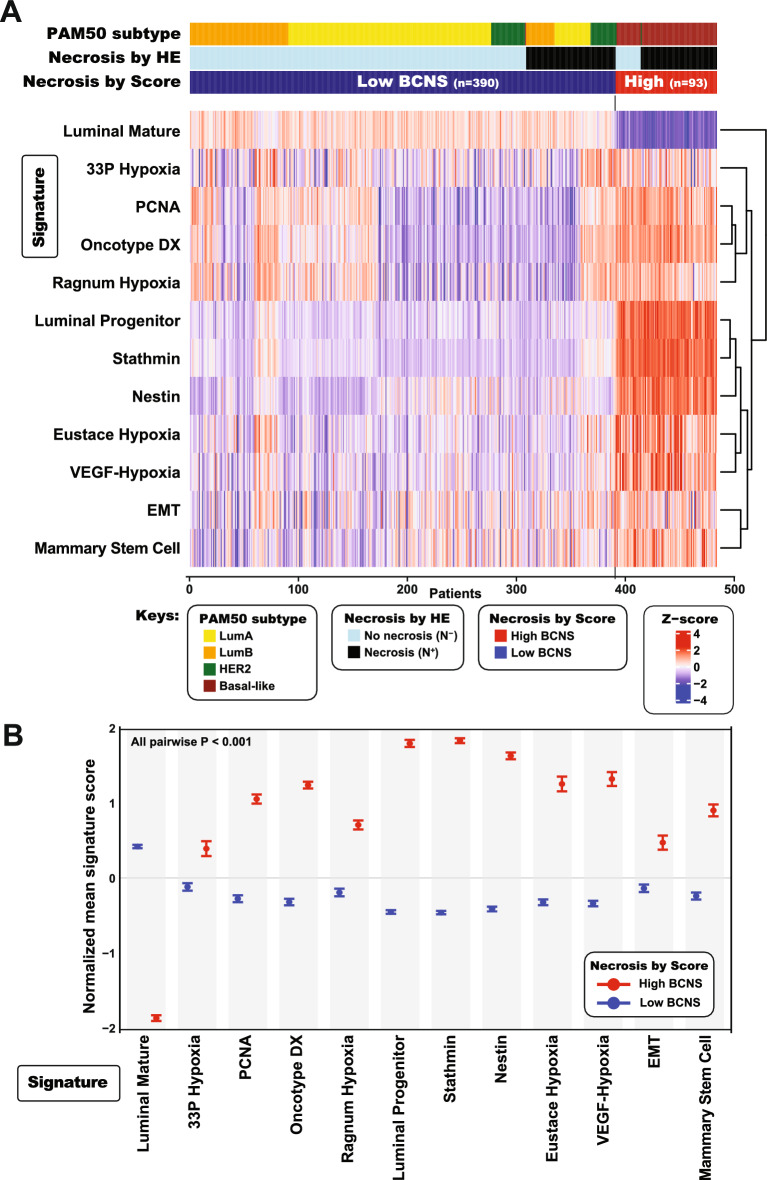
**The Breast Cancer Tumor Necrosis Signature (BCNS) score and relation to basal subtype, plasticity, stemness, proliferation and hypoxic signaling.** (**A**) Heatmap display of signature scores related to plasticity, stemness, proliferation and hypoxia, and its association to the high or low Breast Cancer Necrosis Signature score. (**B**) Error-barplots of signature scores in A (normalized mean z-scores) comparing expression level in relation to necrosis, presented as 95% confidence interval of the mean with p-values evaluated by Mann–Whitney U-test. Data from TCGA, n = 483^26^.

To investigate the relation between the high BCNS score and these activated pathways, we aligned our Necrosis score to multiple gene expression signatures involved in selected transcriptional programs with potential roles in necrosis (Fig. 3). The resulting supervised heatmap showed that these signatures clustered together by expression in relation to the necrosis score and molecular subtypes, with 11/12 showing positive correlation to the BCNS score (Fig. 3A). In addition, all the signatures displayed significant different score levels in error-bar plots when compared to a BCNS score dichotomized into high or low levels (all P < 0.001, Fig. 3B). The different signatures applied in this study represent unique insights into various pathways, and systematic pairwise comparison of gene lists revealed little overlap amongst them (measured by Jaccard Index, Supplementary Figure S1B).

As observed from Fig. 1B and Fig. 3, the HER2 status seemed to influence the BCNS-low cases. Due to the lack of availability of the finer HER2-mapping (evaluated as 0, 1 + , 2 + or 3 +) we utilized the TCGA-HER2-index score^47^ as a pseudo-marker for HER2 to investigate its relationship with our necrosis score. The results showed a significant association between the BCNS and the TCGA-HER2-index in all subtypes—except the basal-like subtype (Supplementary Figure S5), although precautions should be taken when extrapolating HER2 morphological status to this HER2-index score developed from gene expression data.

We followed up the “discordant samples” from Figs. 1B and 3A, where the morphologic necrosis assessment did not match the BCNS score. Sorted by the BCNS score, we still found that the necrosis score we developed captures nuances in expression of the panel of signatures, only in part driven by key markers (ER, PR, HER2) (Supplementary Figure S2B).

### Tumors with necrosis reflect processes linked to proliferation

From GSEA, proliferation was top-ranked and significantly enriched in tumors with necrosis. Indeed, several of the proliferation-related gene sets enriched in breast cancer with necrosis were related to chromosome segregation, organization, condensation, and localization. Gene ontology results reflected proliferation-related processes like cell-cycle phase transition, cytokinesis, membrane fission, and microtubule cytoskeleton organization were enriched in tumors with necrosis. Adding to this, gene sets reflecting E2F targets, involved in regulating the progression of the cell cycle, were also enriched in tumors with necrosis (Supplementary Table S7).

**Fig. 4:**
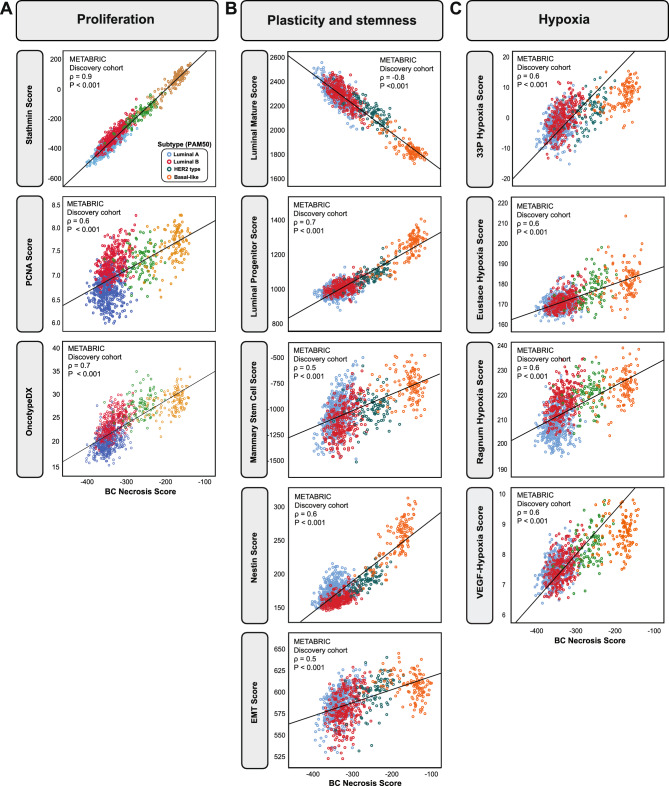
**Relation of the BCNS score and signatures of proliferation, plasticity and stemness, and hypoxic signaling by molecular PAM50 subtypes.** The BCNS score compared to: (**A**) Proliferation signatures of Stathmin, PCNA, and Oncotype DX related to the BCNS score. (**B**) Signatures related to plasticity, stemness and EMT. (**C**) Hypoxia-related signatures related to the BCNS score. Data from METABRIC discovery cohort, n = 939^27^. Scatter plots are presented with p-values by Spearman´s rank correlation and the associated coefficients (ρ).

To explore the relation between proliferation and necrosis further, we examined how independent proliferation signatures associated with tumor necrosis and found higher Stathmin^33^, Oncotype DX^34^ and PCNA scores^35^ in tumors with necrosis, supporting increased tumor cell proliferation in breast cancer with tumor necrosis (TCGA; all P < 0.001, Fig. 4A). These results validated in the METABRIC cohorts, as the BCNS score again was significantly correlated with the Stathmin-, Oncotype DX-, and PCNA scores (all P < 0.001, ρ = 0.60–0.74) and displayed a necrosis-signature relationship also observable within the four molecular subtypes (Fig. 4A and Supplementary Figure S6A).

### Necrosis associates with elevated plasticity and stemness properties

Validating and underscoring the link between breast cancer histopathological necrosis and BCNS, tumor plasticity and the basaloid, stem-like features, we demonstrated a negative correlation between the BCNS score and a mature luminal cell score, and strong positive correlations between BCNS score and signatures reflecting EMT, stemness and progenitor features in the METABRIC cohorts (Fig. 4B and Supplementary Figure S6B). These results supported the notion of a high BCNS score and its relation to the basal-like subtype described earlier.

### High BCNS score points to increased hypoxia signaling

Necrosis is closely linked to hypoxia, and this was further supported by our finding of gene sets reflecting hypoxia enriched in tumors with necrosis (Supplementary Table S7). We therefore investigated the relation between the BCNS score and multiple independent hypoxia signatures (see Materials & Methods and Fig. 3). We validated strong positive correlations between the BCNS score and the hypoxia scores, across several different hypoxia signatures in the METABRIC cohorts, and found concurrent high BCNS score and hypoxia scores in the basal-like subtype, supporting that these tumor features are related and have a role in the elevated tumor aggressiveness we observe (Fig. 4C and Supplementary Figure S6C).

### Mutational patterns in relation to tumor necrosis

**Fig. 5:**
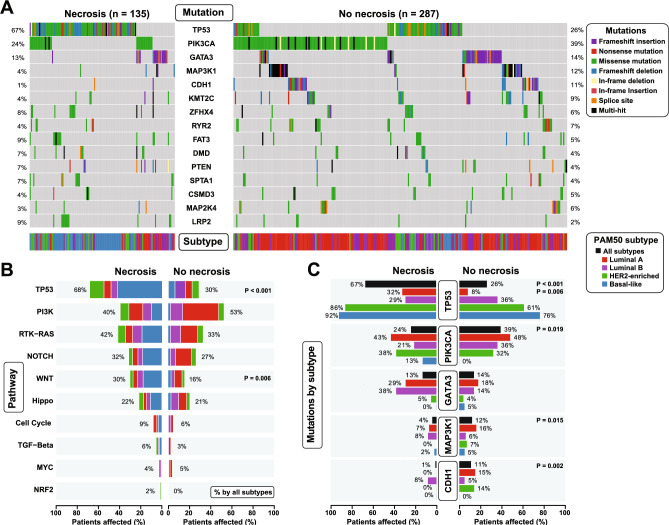
**Mutational patterns by morphologic necrosis status.** (**A**) Mutational patterns in tumors with and without necrosis evaluated by the 15 most frequently mutated genes among 422 female BC patients. (**B**) Differences in oncogenic pathways in tumors based on PAM50 subtypes and morphologic necrosis status. (**C**) Mutational differences in top five mutated genes by PAM50 subtype and necrosis status. Mutation data from TCGA BRCA study^26^.

When investigating somatic gene mutations in relation to tumor necrosis, we found different mutational patterns in tumors with (n = 135) or without necrosis (n = 287) (Fig. 5). The overall mutation burden was higher in patients with necrotic tumor tissues compared to non-necrotic tumor tissues (all variants, mean of 84.8 and 60.9 mutations per sample, respectively). In tumors with necrosis, we found that *TP53* was the most frequently mutated gene (overall cohort mutation frequency of 39%, with 67% in tumors with necrosis and in 26% of tumors without necrosis; Fig. 5A). Similarly, 39% of non-necrosis tumors had a *PIK3CA* mutation compared to 24% of the tumors with necrosis (overall mutation rate of 35%). These results were supported by pathway analysis confirming that the most enriched pathways in tumors with necrosis were the TP53 (affecting 68% of cases, P < 0.001) and WNT pathways (P = 0.006). In tumors without necrosis, the Phosphatidylinositol-3-Kinase (PI3K) pathway was the most mutated pathway at 53% of cases, although not statistically significant (P = 0.164) compared to the necrotic samples. Together, these results suggest that necrosis positive tumors endorse different pathways compared to tumor tissues without necrosis (Fig. 5B).

Indeed, a closer investigation of the mutational differences by necrosis revealed differences among top five most frequently mutated genes and within PAM50 subtypes (Fig. 5C). *TP53*, *PIK3CA*, *MAP3K1* and *CDH1* were significantly different in mutation rate of N^+^ and N^-^ tumors (all P ≤ 0.019), but not *GATA3*. At the subtype-level, *TP53* had a higher mutation rate in the necrosis positive cases in the Luminal A (PAM50) subgroup (32% vs. 8%; P = 0.006). Other differences within the PAM50 subgroups were observed (although not significant) including higher level of *GATA3* mutations in the HER2-enriched subtype (38% vs. 14% in tumors with and without necrosis), and the apparent lack of *CDH1* mutations in necrosis tumors (0–1%, all subtypes except Luminal B), compared to overall 11% in non-necrosis tumors. There was no apparent difference in hotspot distribution across mutations in the five top mutated genes (*TP53*, *PIK3CA*, *GATA3*, *MAP3K1* and *CDH1*) at the corresponding protein sequences, comparing the necrosis versus no necrosis (Supplementary Figure S7).

## Discussion

The presence of morphologic tumor necrosis has previously been demonstrated to be associated with aggressive tumor features and reduced survival in breast cancer^13–15,49^. In our study, we found morphologic tumor necrosis associated with molecular features like ER- and PR negativity, HER2 positivity, and the basal-like subtype of breast cancer. On this background, we aimed at further elucidating the underlying biology of necrosis by investigating gene expression alterations in breast cancer with the use of global transcriptomic data readily available from two public repositories – the TCGA, and two independent METABRIC cohorts^26,27^.

By integrating gene expression data and tissue-based breast cancer necrosis information, we obtained a novel Breast Cancer Necrosis Signature (BCNS) score that demonstrated associations with aggressive tumor features and independent prognostic value, also in the subset of patients with hormone receptor positive luminal tumors, pointing to an added value of the BCNS score to the PAM50 algorithm as prognosticator. To our knowledge, our study is the first to describe tumor necrosis-related alterations of gene expression in breast cancer, and the first to construct a necrosis-related signature with clinical relevance in this cancer type. However, in endometrial cancer, a similar study was performed by Bredholt *et al.*, identifying a tumor necrosis signature score highlighting an association between a high necrosis score and reduced survival^50^, in support of our data. We were unable to directly compare the signatures derived by us and Bredholt *et al. *due to deviating probes/genes between the datasets used in the respective studies. The BCNS score allows for a deeper insight into the biology related to morphologic necrosis and is therefore an important addition to understanding necrosis and its relation to aggressive BC.

Perou *et al.* were the first to describe the intrinsic molecular subtypes of breast cancer, based on gene expression clusters^51,52^. Later, individual and combined immunohistochemical surrogate markers have been suggested to predict the basal-like phenotype, without full consensus being reached^53^. In our analyses, morphologically evaluated breast cancer necrosis strongly predicts the basal-like phenotype. Intriguingly, the BCNS score derived from mRNA expression data predicts this phenotype even stronger, also in multivariate analyses, suggesting that the BCNS score could be a valid marker identifying the basal-like phenotype of breast cancer, and also capturing aggressive BC features across subtypes.

Further, we demonstrate strong correlations between the BCNS score and gene expression programs of progenitor features, plasticity, and EMT (by both GSEA and signature score analyses), supporting a link between breast cancer necrosis and basal- and stem-like features. Indeed, studies has linked EMT to the basal-like phenotype, by immunohistochemical staining, cell experiments and gene expression analyses^54,55^. Also, stemness is found at highest rate in the basal-like subtypes of breast cancer, negatively associated with immune activation, highlighting the complexity and interactions among pathways, regulated at the tumor microenvironment level^56–58^. Based on this, we suggest future work to include an independent validation of the BCNS score as predictor of the basal-like phenotype, aligning it with *BRCA1* germline mutations and comparing the BCNS score to the PAM50 prediction algorithm and other markers of this molecular phenotype.

Proliferation is a required process in cancer development and progression^59,60^. We found three independent proliferation-related scores (Stathmin, PCNA and Oncotype DX) to be increased in tumors with necrosis. A link between necrosis and proliferation has been confirmed in endometrial cancer patient samples^50^, and also experimentally by *in vitro* and *in vivo* models in triple-negative breast cancer^61^, but is to our knowledge not previously demonstrated in tissue-based analyses in breast cancer. Again, the elevated proliferation score in the necrosis positive samples falls perfectly within the increased aggressiveness we observe in these tumors.

Hypoxia is a hallmark of cancer^60^, viewed as a consequence of rapid tumor growth and accompanying inadequate blood supply^62^. Cancer hypoxia has pivotal roles in metabolic reprogramming, stem cell signatures, angiogenesis, extracellular matrix organization and metastasis^60,63–66^. Our data point to significant associations between the presence of tumor necrosis and gene expression programs reflecting hypoxia, advocating higher levels of hypoxia in breast cancer with necrosis. We also found strong positive correlations between high levels of the BCNS score and several signature scores reflecting hypoxia in independent data sets. We relate this to the previously mentioned study on endometrial carcinomas, which also demonstrated associations between tumor necrosis and transcriptome signatures representative of hypoxia as well as relations to angiogenesis and inflammation^50^. Hypoxia plays an important role in tumor progression, metastasis, and as an inducer of epithelial-to-mesenchymal transition (EMT)^58,67^, processes likely contributing to the increased aggressive tumor features and poor survival we see in breast cancer patients with tumor necrosis and a high BCNS score. However, although targeting hypoxia appears as an attractive therapeutic target, drug failure by developing treatment resistance is common^68^.

The signatures of *stathmin*, *luminal mature*, *luminal progenitor* and *nestin* scores were the top four signature scores that are strongest associated with the basal-like subgroups, with all but the luminal mature signature demonstrating positive correlations. As single markers, both Stathmin and Nestin has been shown to associate with the basal-like phenotype, *BRCA1* mutations and aggressive tumors^33,44^, underscoring the current knowledge that the basal-like subtype is particularly aggressive and therefore aligning with the BCNS score. In contrast, within Luminal B, drivers of the BCNS score appeared to be the signatures of *stathmin* and *luminal mature*, underscoring that the BCNS captures biological aspects beyond just subtypes.

In line with the observation of aggressive clinical features for tumors with necrosis, we found that mutations in the TP53- and WNT pathways were significantly enriched in tumors with necrosis (68% and 30% cases affected). But we also noted the high mutation frequency of the PI3K pathway (53% of cases) in the non-necrosis tumors. Concomitant with this, we find a higher mutation frequency of TP53 in the necrosis positive group, and a high PI3K-related mutation rate in *PIK3CA*, *PTEN* and mitogen-activated protein kinases *MAP3K1* and *MAP2K4* in the non-necrosis group, with possible implications for treatment^69,70^. Although these findings were colored by the molecular subtypes of breast cancer with TP53 most often being mutated in the HER2- and Basal-like subtypes^26^, notable differences in mutation frequencies were observed in their mutation profiles, suggesting differences in their cancer genome profiles comparing necrosis positive versus non-necrosis tumors. In fact, survival differences occur if one compares patients with TP53 mutated and PI3K mutated breast cancer, as the latter group has better survival probability, concurring with luminal subtypes. However, as far as we know, there is no literature describing mutational alterations in relation to necrosis, and no necrosis-specific driver gene has been identified.

ER and PR are well recognized as key drivers of breast cancer phenotypes, and our data suggest that HER2 may similarly influence the BCNS signature. The observed correlation between BCNS and both the histopathological HER2 status and the TCGA-HER2-index in Luminal A, Luminal B, and HER2-enriched tumors – but not in Basal-like tumors – supports the notion that HER2 signaling could act as a driver of necrosis-associated biology in specific molecular subgroups. Although high BCNS scores are strongly enriched in the Basal-like subtype, this does not simply reflect confounding by intrinsic subtype classification. The BCNS captures necrosis-associated biology that extends beyond conventional subtype markers, as demonstrated by its correlations with HER2 indices in non-Basal-like groups and by the heterogeneous distribution of Luminal B tumors along the BCNS ranking. Thus, the score appears to provide additional, biologically relevant information rather than merely mirroring established subtype definitions.

The BCNS score and morphological necrosis capture related but distinct aspects of tumor biology; the former reflects transcriptional activity, while the latter is a binary histological readout. Accordingly, some discordance is expected. However, the benefit of the BCNS lies in providing a more continuous and systemic measure of necrosis-associated processes, again supporting the view that BCNS complements rather than duplicates morphological scoring.

Currently, there are no drugs that specifically targets necrosis. Treatment strategies aimed at necrosis-associated pathways could help, but poor vascular supply in necrotic tumors limits the effect of treatment provided systemically, and chemotherapy agents may themself induce necrosis or induce hypoxic conditions making tumors with necrosis challenging to treat. One approach to alleviate the effects of necrosis might therefore be through normalizing the vasculature through anti-angiogenic agents (e.g. targeting VEGF receptors with Bevacizumab)^71^. Such an approach is also supported by the very recent study by Perou & Olopade, finding a 13-gene VEGF-hypoxia signature to be upregulated in Basal-like and necrotic tumors in women of African origin^43^. Future methods may also include a more mechanistic approach, by targeting peri-necrotic tumor zones by using hypercin-linked nanoparticles for now only tested in preclinical-models^72^.

We have used public data that is followed by some limitations. Firstly, the original studies were designed to make use of bulk tissue data for preparing microarray data and studying necrosis-related morphology. A refined study design employing micro-dissected tissue sections for the mRNA analyses could have provided more information about each of the tumor compartments and their interactions than we have been able to demonstrate^42^. Secondly, our exploratory and descriptive approach is not sufficient to differentiate between the causes and consequences of tumor necrosis, as we have not followed up with functional validation. However, a strength of our analyses is the use of multiple independent datasets to evaluate the biological processes of tumor necrosis. Also, we consider our signature approach as reasonable and sufficiently powerful to detect new biomarkers, as this strategy potentially adjusts for a study design lacking spatial information^73^.

## Conclusion

By integrative approaches, we have demonstrated that breast cancer necrosis is associated with aggressive tumor features, pointing to the basal-like phenotype and stem-like features, hypoxia, increased proliferation, and reduced survival, considered unfavorable in breast cancer. The novel Breast Cancer Necrosis Signature (BCNS) score is a strong predictor of the basal-like phenotype and a strong prognosticator in breast cancer. The signature comprising the BCNS are representative of genes known to be involved in breast cancer and are particularly associated with estrogen-related signaling. Further validation of our results is required to finally conclude whether the BCNS score may contribute with added value in the clinical setting of breast cancer diagnosis and management.

## Supplementary Information

Supplementary Information 1.

Supplementary Information 2.

## Data Availability

The underlying findings in this study are based on data generated by the TCGA Research Network^26^ (https://www.cancer.gov/tcga), Thennavan^28^ and the METABRIC study^27^.
