# Supplementary material for: Tumor necrosis associates with aggressive breast cancer features, increased hypoxia signaling and reduced patient survival
Source: Sci Rep. 2025 Nov 27;15:45518. doi: 10.1038/s41598-025-29905-3 (PMC12749603; doi:10.1038/s41598-025-29905-3)
Supplement: Supplementary file 1 — Supplementary Information 1. [file 41598_2025_29905_MOESM1_ESM.pdf]

SUPPLEMENTARY MATERIAL:

Title: Tumor Necrosis Associates with Aggressive Breast Cancer Features, Increased Hypoxia Signaling and Reduced Patient Survival

Authors: Astrid A. Syrtveit[1], Lise M. Ingebriktsen[1,†], Amalie F. Tegnander[1,†], Lars A. Akslen[1,2], Elisabeth Wik[1,2,‡] & Erling A. Hoivik[1,2,‡,*]

Affiliations

[1]Centre for Cancer Biomarkers CCBIO, Department of Clinical Medicine, Section for Pathology, University of Bergen, Norway

[2]Department of Pathology, Haukeland University Hospital, Bergen, Norway

[†]These authors contributed equally to this work

[‡]These authors jointly directed this work

***Corresponding author:** erling.hoivik@uib.no

Content:

- Supplementary Figures S1-7.
- Supplementary Tables 1-7.
- Supplementary Data File.

Supplementary Figure S1

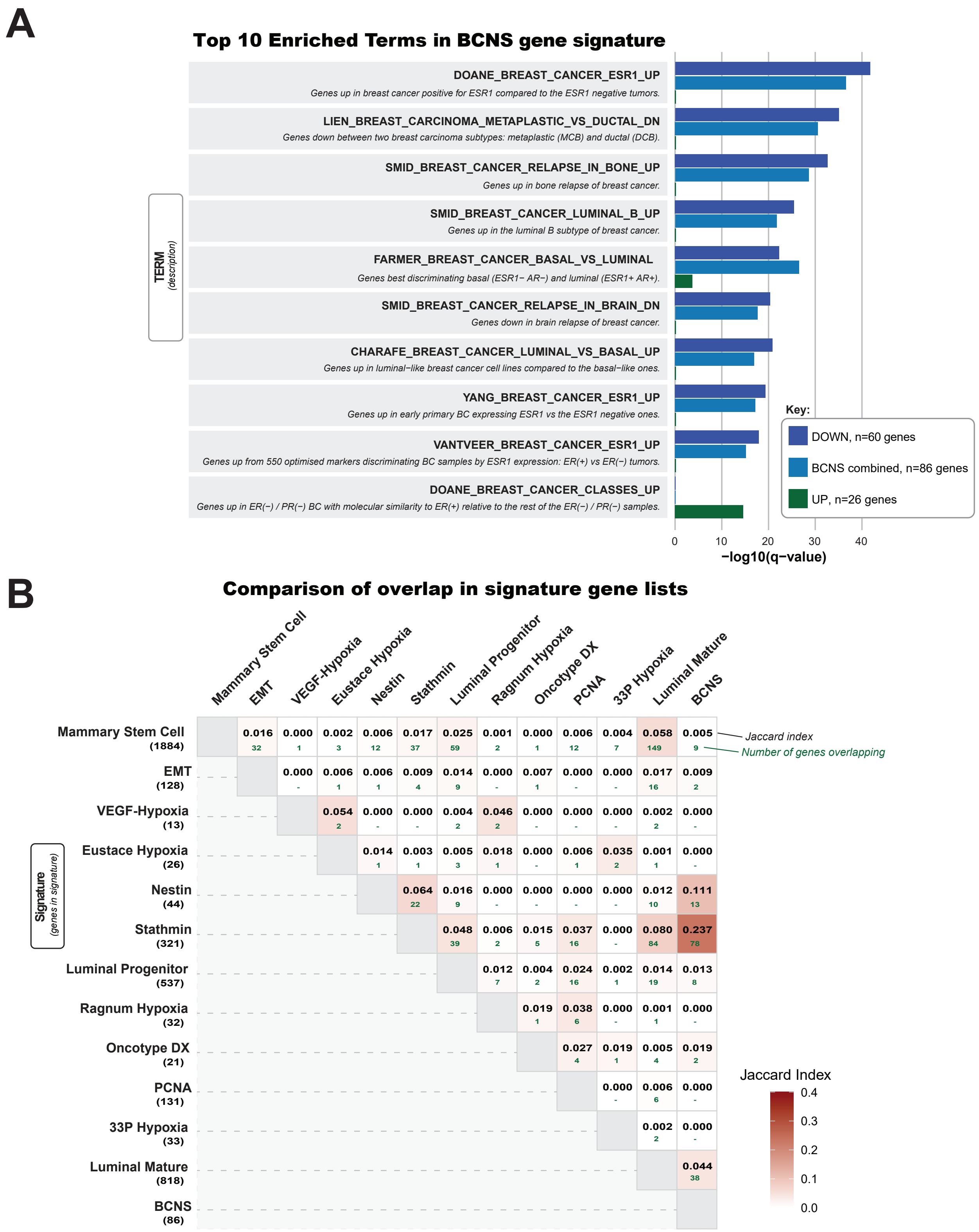

Supplementary Figure S1: Functional enrichment analysis of the BCNS gene list and overlap with other gene signatures. (A) Results of the ten most enriched terms of the BCNS gene list (DOWN, UP or combined) related to the related to functional enrichment *(over-representation analysis)* from MSigDB using oncoErichR [32]. All results were from the '*CHEM_GEN_PERTURB*' category. Results are dominated by the *breast cancer* term with descriptions related to estrogen-related signaling. **(B)** Display of pairwise interactions among the genes in the signatures used in this study by Jaccard Index. The Jaccard Index (range 0-1) indicates the similarities among the signatures compared (0-100% overlap). Please note that the higest Jaccard Index is at 0.237 (BCNS compared to Stathmin), suggesting little overlap in general, among the gene lists in the signatures applied in this study. Number of genes comprising the full signatures, and number of overlapping genes in pairwise intersections are indicated. The *Symbol* annotation of genes were used in the comparisons.

Supplementary Figure S2

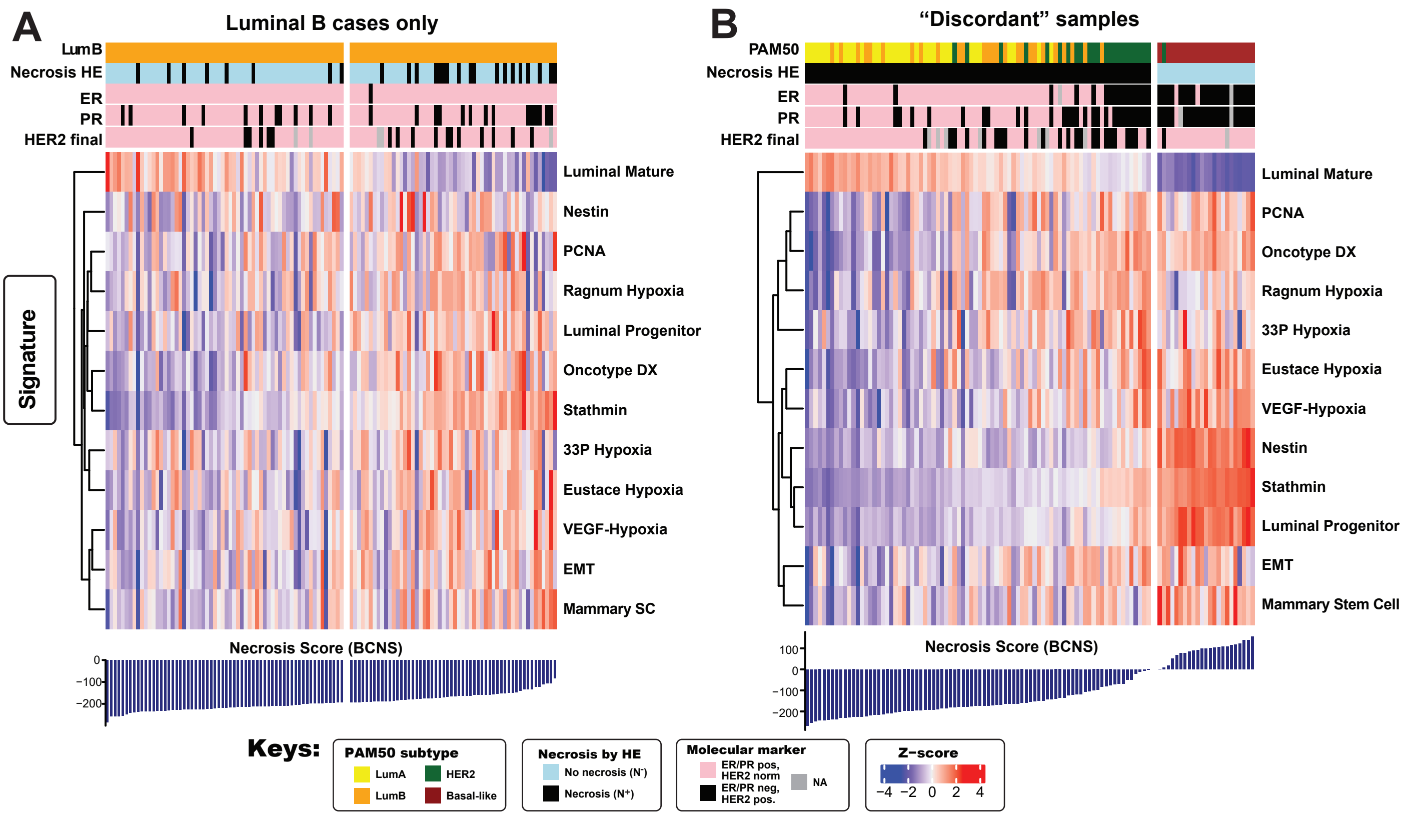

Supplementary Figure S2: Subset clustering of Luminal B cases and BCNS/HE-discordant samples. (A) Clustering of the panel Luminal B cases only from Figure 1B, with signature scores. Data are sorted by BCNS score, and show two clusters defined by mean value of BCNS. (B) Clustering of BCSN score and HE-necrosis “discordant” samples extracted from Figure 3A, sorted by BCNS score and HE-necrosis status (absent/present). Data from TCGA [26].

Supplementary Figure S3

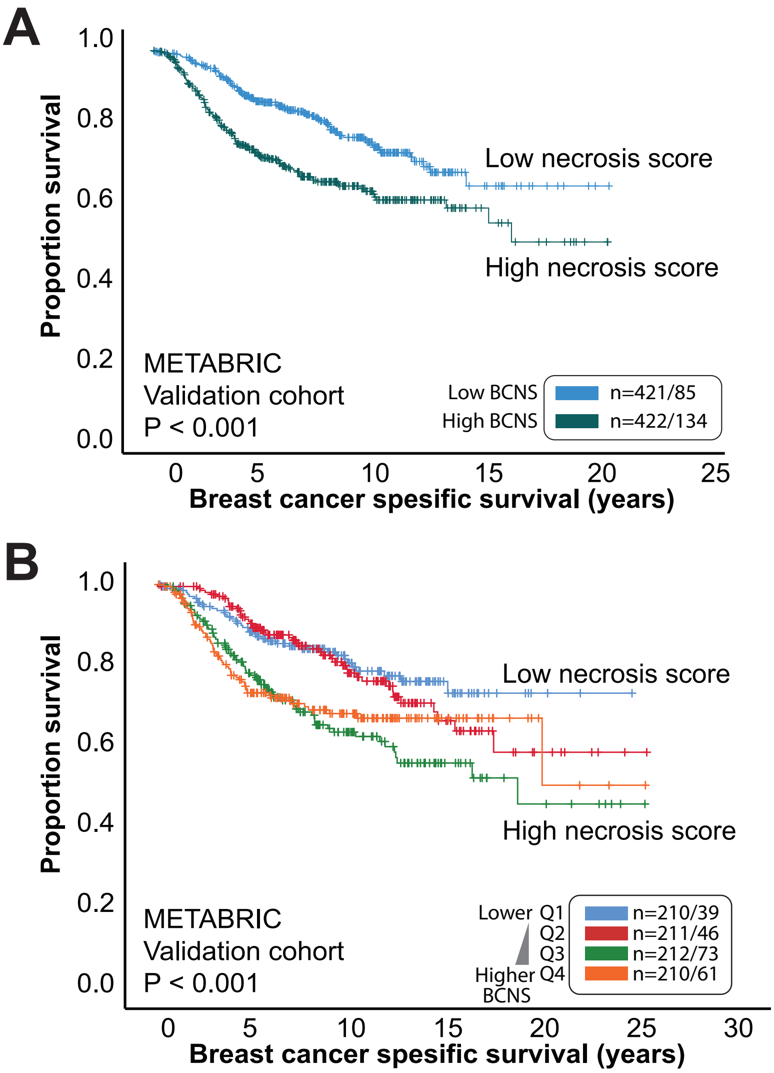

Supplementary Figure S3 : Validation of the BCNS score in relation to survival. A high necrosis score associated with shorter disease-specific survival in the METABRIC validation cohort as evaluated by low-high scores **(A)** and by quartiles Q1-Q4 **(B)**. METABRIC cohort, n=845 [27]. Number of cases and events are indicated in the Kaplan-Meier survival curves.

Supplementary Figure S4

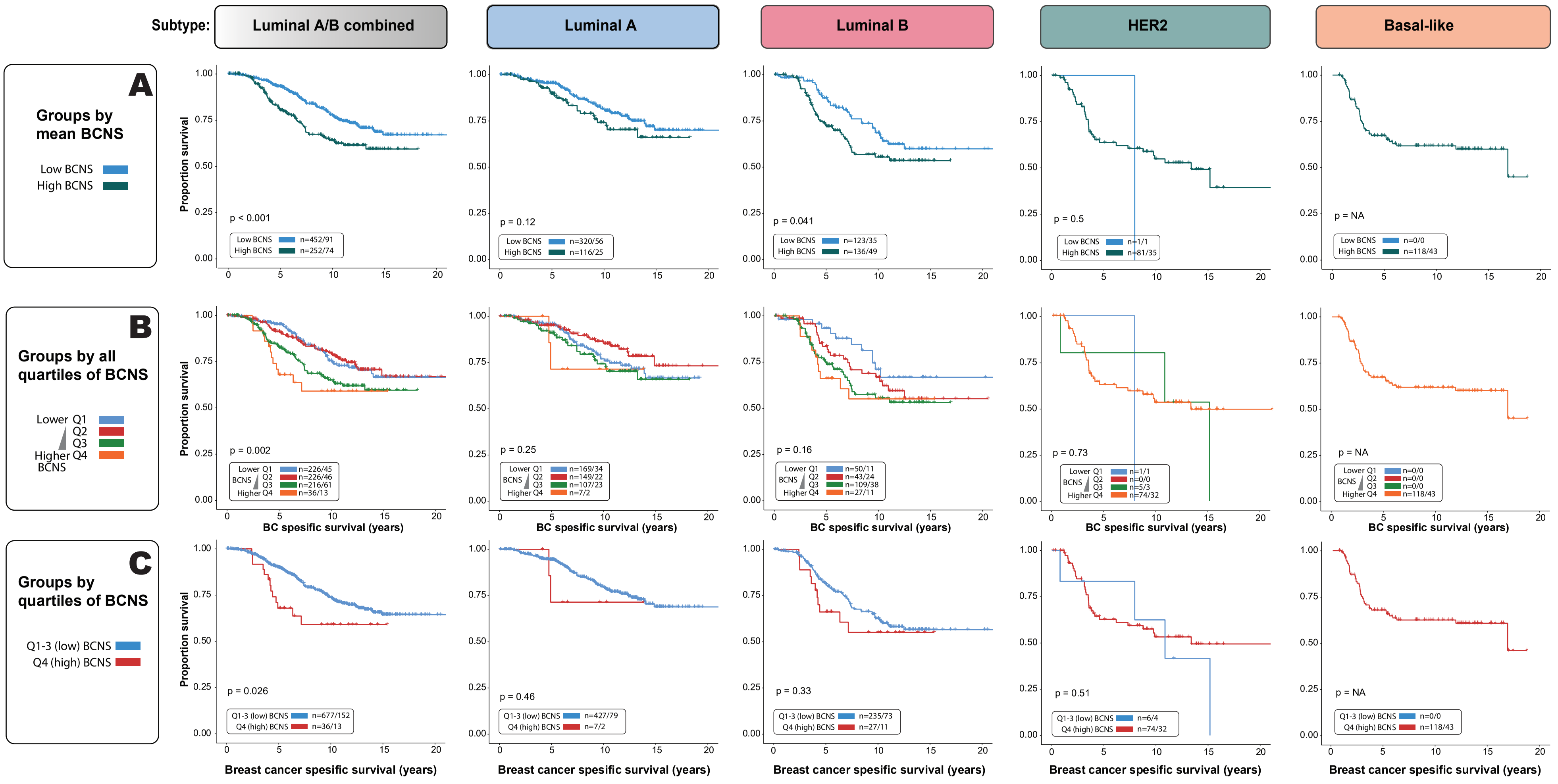

Supplementary Figure S4: The BCNS score in relation to survival across PAM50 molecular subtypes. BCNS score evaluated through median cutoff **(A)**, by each fur quartiles **(B)** and by upper Q4 quartile **(C)**. A high necrosis score is associated with shorter disease-specific survival in a Luminals subtypes combined (A/B) and Luminal B subtype alone, but not with Luminal A, HER2 or Basal-like subtypes. Please note the lack of cases for subtypes HER2 and Basal-like for some of the groups tested in survival analysis. All cases for the Basal-like group is allocated to the BCNS-high groups (above-mean, or quartile Q4). Number of cases/events are indicated in the Kaplan-Meier survival curves. All data from the METABRIC Discovery cohort, n= 939 [27].

Supplementary Figure S5

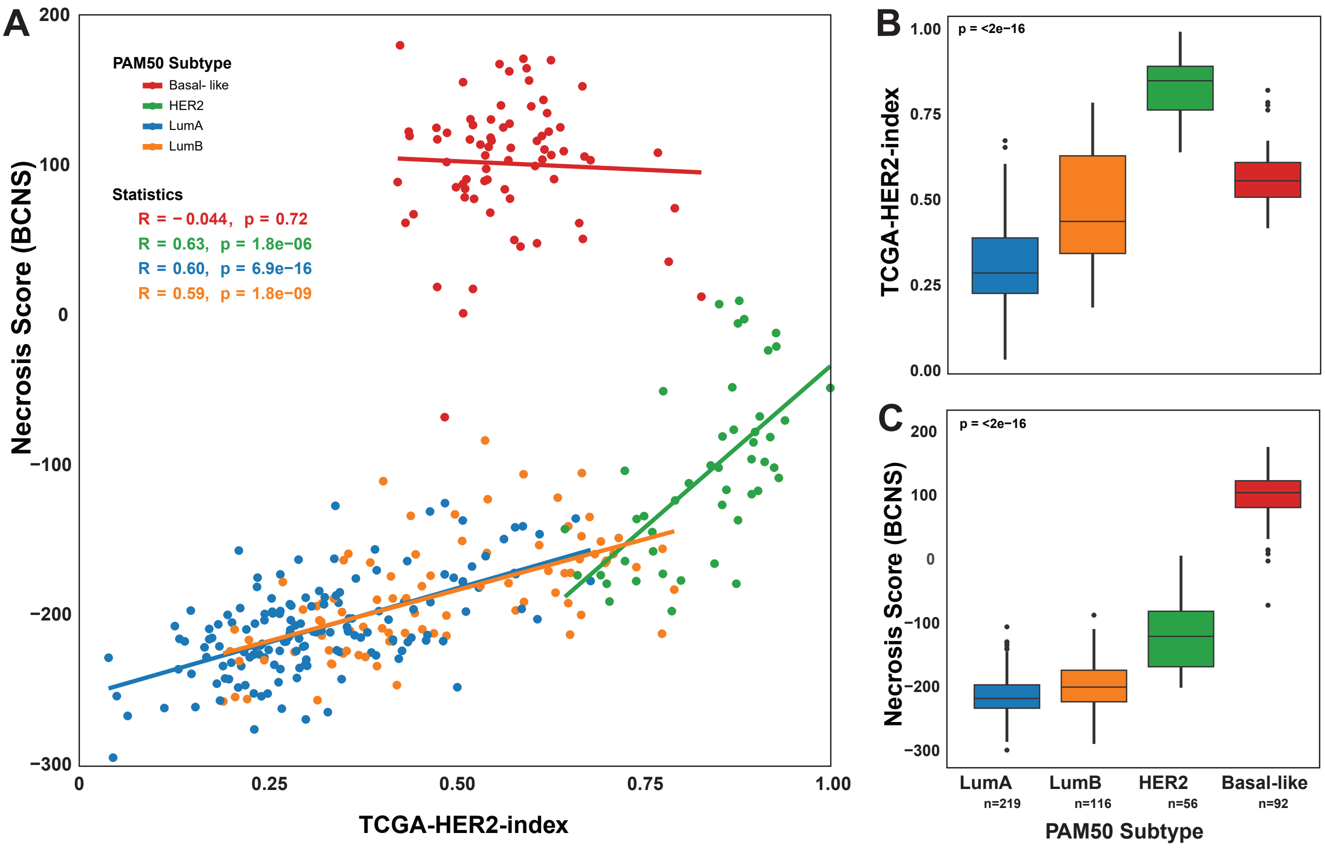

Supplementary Figure S5: The Necrosis score in relation to HER2 using an index score. (A) A scatter plot of the Necrosis (BCNS) score in relation to the TCGA-HER2-index score. Boxplot by PAM50 molecular subtype by the TCGA-HER2-index score **(B)** and towards the BCNS score **(C)**. The TCGA-HER2-index score (weighted from n=1818 genes) was developed by Li *et. al.*, from an TCGA pan-cancer approach, where higher index corresponds to a closer connection to HER2-enrichment in the tumor [47]. The results indicates that the BCNS score is not driven by HER2 for the Basal-like subtype.

Supplementary Figure S6

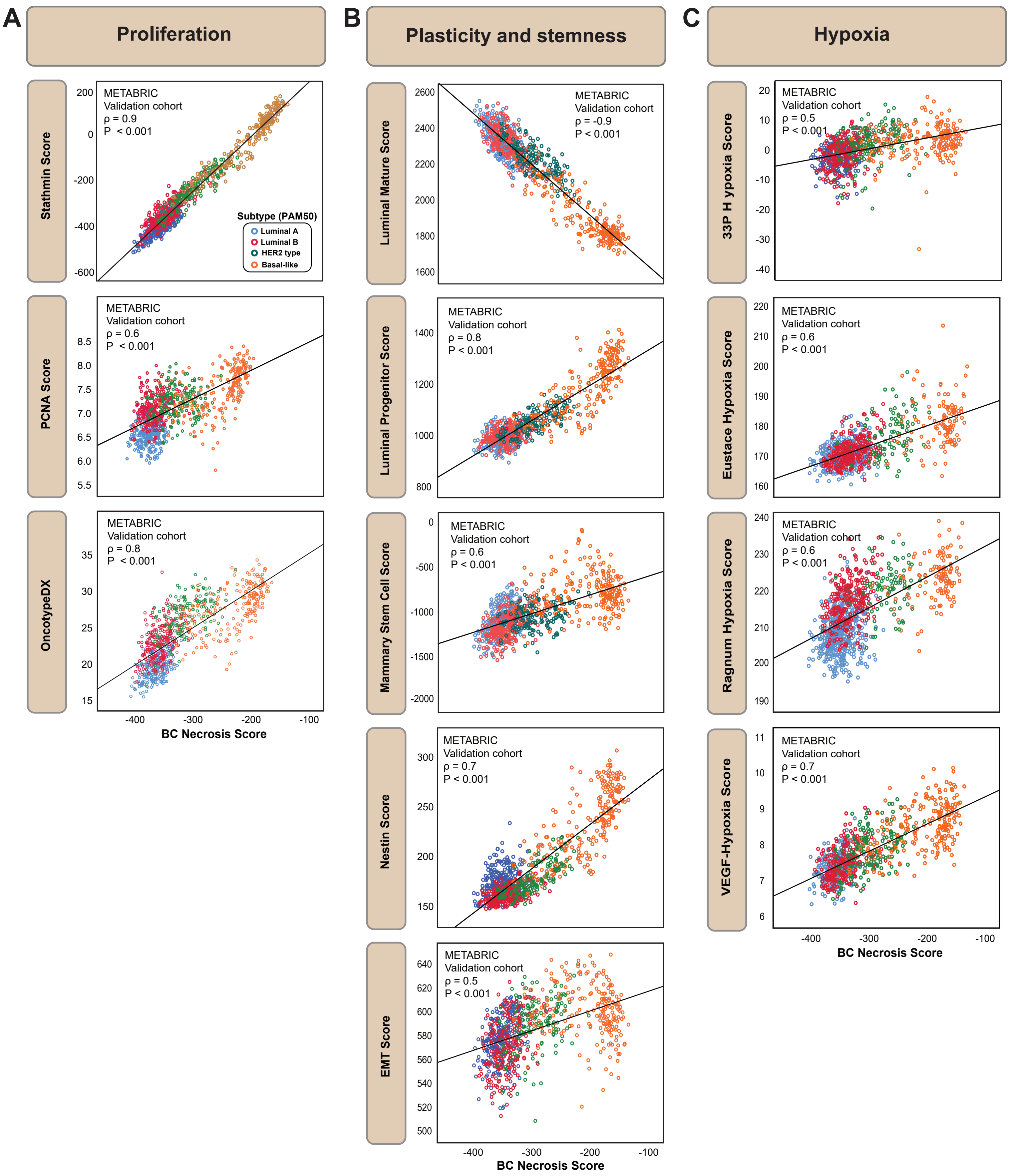

Supplementary Figure S6: Validation of the relations of BCNS score to signature scores of plasticity, stemness, proliferation and hypoxia signaling by molecular subtypes. The BCNS score compared to: **(A)** Proliferation signatures of Stathmin, PCNA, and Oncotype DX. **(B)** Signatures related to plasticity, stemness and EMT. **(C)** Hypoxia-related signatures. Data from METABRIC validation cohort, n=845 [27]. Scatter plots are presented with p-values by Spearman´s rank correlation and the associated coefficients (ρ)

Supplementary Figure S7

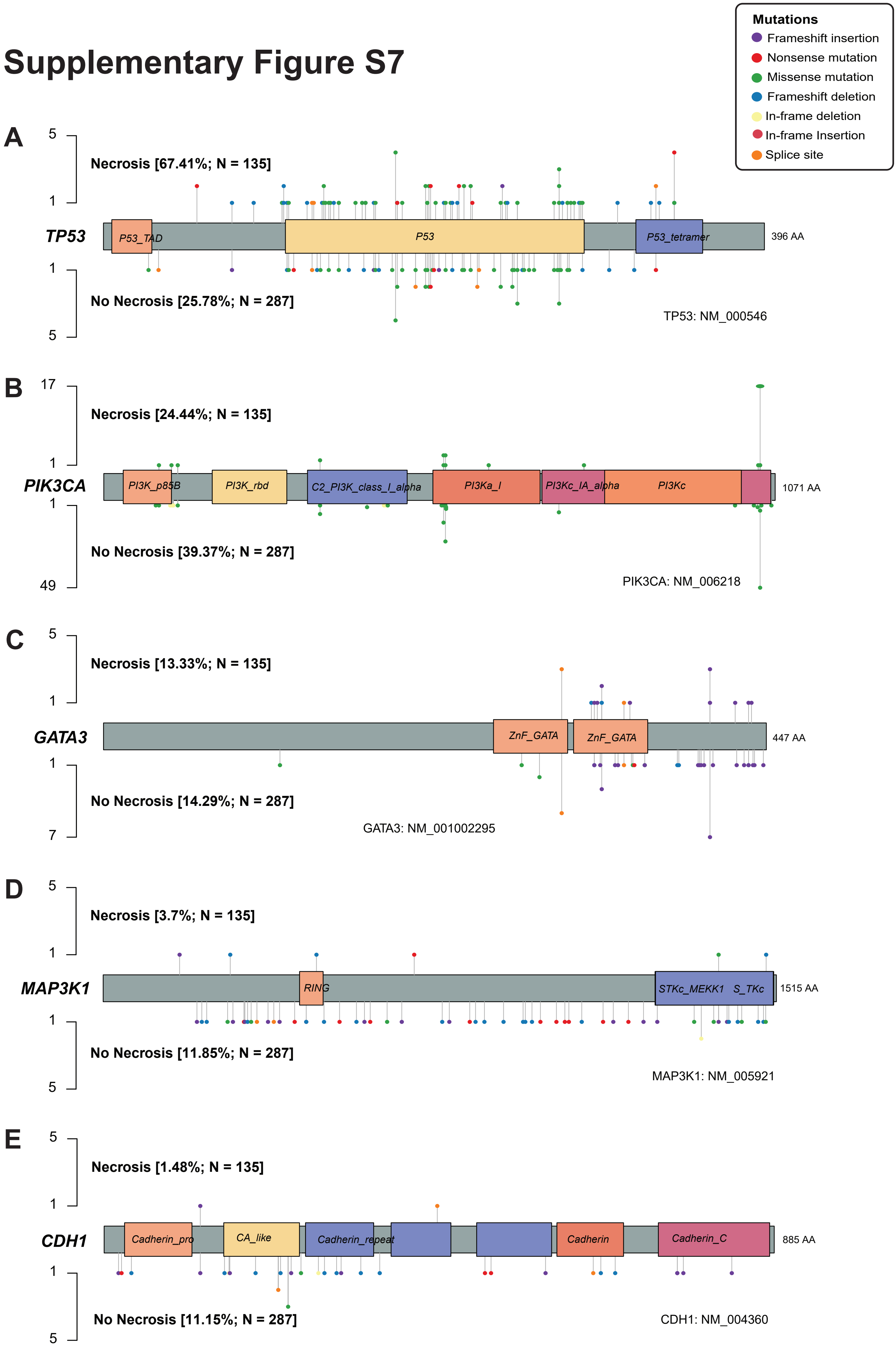

Supplementary Figure S7: Lollipop plot of top five selected genes by corresponding protein mutations. Top-five most frequently mutated genes in the TCGA dataset [26], showing mutations, including hotspots on protein structures comparing necrotic and non-necrotic tumors at corresponding protein structures (domains indicated). *TP53* **(A)**, *PIK3CA* **(B)**, *GATA3* **(C)**, *MAP3K1* **(D)**, and *CDH1* **(E)**. Number of mutations at indicated protein amino acid (AA) positions are indicated by height of lollipops according to individual scales to the left, note differences on axes.

Supplementary Table S1.

Contingency table of dichotomized BCNS-score towards morphologic necrosis.

Morphologic necrosis (number of cases)	BCNS category (number of cases)		
	**High-BCNS**	**Low-BCNS**	**Sum**
**Necrosis absent (N-)**	23	308	331
**Necrosis present (N+)**	70	82	152
**Sum**	93	390	483

Statistics: Pearson's Chi-squared test with Yates' continuity correction; p-value < 2.2e-16.

Supplementary Table S2.

Associations of Breast Cancer Necrosis Signature (BCNS) Score by quartiles (Q) and clinico-pathologic and molecular markers in METABRIC Discovery and Validation cohorts.

A) METABRIC Discovery cohort (n = 939)

	Breast Cancer Necrosis Signature (BCNS) score				
**Variables**	**Q1 n (%)**	**Q2 n (%)**	**Q3 n (%)**	**Q4 n (%)**	**P-value**
**Tumor size**					0.07
≤ 20mm	108 (46.2%)	104 (44.3%)	101 (43.0%)	96 (40.9%)	
> 20mm	126 (53.8%)	131 (55.7%)	134 (57.0%)	139 (59.1%)	
**Histologic grade**					**<0.001**
Grade 1/2	181 (77.7%)	150 (63,8%)	96 (40.9%)	28 (11.9%)	
Grade 3	52 (22.3%)	85 (36.2%)	139 (59.1%)	207 (88.1)	
**Nodal status**					**<0.001**
Negative	140 (59.8%)	134 (57.0%)	109 (46.4%)	99 (42.1%)	
Positive	94 (40.2%)	101 (43.0%)	126 (53.6%)	136 (57.9%)	
**ER status**					**<0.001**
Positive	232 (99.1%)	232 (98.7%)	229 (97.4%)	63 (22.8%)	
Negative	2 (0.9%)	3 (1.3%)	6 (2.6%)	172 (73.2%)	
**Molecular subtype (PAM50)**					**<0.001**
Luminal A	181 (77.4%)	160 (68.1%)	116 (49.4%)	9 (3.8%)	
Luminal B	52 (22.2%)	75 (31.9%)	114 (48.5%)	27 (11.5%)	
HER2-enriched	1 (0.4%)	0 (0.0%)	5 (2.1%)	81 (34.5%)	
Basal-like	0 (0.0%)	0 (0.0%)	0 (0.0%)	118 (50.2%)	

B) METABRIC Validation cohort (n = 845)

	Breast Cancer Necrosis Signature (BCNS) score				
**Variables**	**Q1 n (%)**	**Q2 n (%)**	**Q3 n (%)**	**Q4 n (%)**	**P-value**
**Tumor size**					**0.02**
≤ 20mm	93 (44.1%)	95 (45.0%)	68 (32.1%)	91 (42.9%)	
> 20mm	118 (55.9%)	116 (55.0%)	144 (67.9%)	120 (57.1%)	
**Histologic grade**					**<0.001**
Grade 1/2	146 (79.3%)	119 (63.0%)	74 (37.6%)	24 (11.9%)	
Grade 3	38 (20.7%)	70 (37.0%)	123 (62.4%)	178 (88.1%)	
**Nodal status**					0.15
Negative	106 (50.2%)	124 (58.8%)	103 (48.8%)	107 (50.7%)	
Positive	105 (49.8%)	87 (41.2%)	109 (51.2%)	104 (49.3%)	
**ER status**					**<0.001**
Positive	198 (97.1%)	198 (97.5%)	163 (80.7%)	27 (13.5%)	
Negative	6 (2.9%)	5 (2.5%)	39 (19.3%)	173 (86.5%)	
**Molecular subtype (PAM50)**					**<0.001**
Luminal A	127 (60.2%)	103 (48.8%)	25 (11.8%)	0 (0.0%)	
Luminal B	79 (37.4%)	90 (42.7%)	55 (25.9%)	0 (0.0%)	
HER2-enriched	5 (2.4%)	15 (7.1%)	98 (46.2%)	35 (16.6%)	
Basal-like	0 (0.0%)	3 (1.4%)	34 (16.0%)	176 (83.4%)	

n=number of patients, Q=quartiles

Supplementary Table S3.

Cox multivariate survival analysis, METABRIC Validation cohort.

The Breast Cancer Necrosis Signature (BCNS) score was included as continuous variable (n=771 with all information included for all cases).

		Unadjusted			Adjusted		
**Variable**	**n (%)**	**HR**	**95% CI**	**P-value**	**HR**	**95.0% CI**	**P-value**
**Tumor size**				**<0.001**			**<0.001**
≤ 20 mm	319 (41%)	1.0			1.0		
> 20 mm	452 (59%)	2.0	1.5-2.7		1.8	1.4-2.5	
**Histologic grade**				**<0.001**			0.2
Grade 1/2	363 (47%)	1.0			1.0		
Grade 3	%	1.7	1.2-2.2		1.2	0.9-1.7	
**Lymph node status**							**<0.001**
Negative	390 (51%)	1.0		**<0.001**	1.0		
Positive	381 (49%)	2.9	2.1-3.8		2.4	1.8-3.2	
**BCNS score**	771 (100%)	1.003	1.001-1.005	<0.001	1.002	1.000-1.004	**0.04**

HR=Hazard ratio, CI=Confidence interval, n=number of patients. Missing cases: Histologic grade, n=73; Tumor size, n=20; Follow-up time, n=2.

Supplementary Table S4.

Cox regression multivariate analysis.

Clinico-pathologic variables and the Breast Cancer Necrosis Signature (BCNS) score were included as categorized variables (by quartiles), in the METABRIC Discovery and Validation cohorts.

A) METABRIC Discovery cohort (n=939)

		**Unadjusted**			**Adjusted**		
**Variable**	**n (%)**	**HR**	**95.0 % CI**	**P-value**	**HR**	**95.0 % CI**	**P-value**
**Tumor size**				**<0.001**			
≤ 20 mm	409 (44%)	1.0			1		
> 20 mm	530 (56%)	2.0	1.5-2.6		1.6	1.2-2.1	**0.002**
**Histologic grade**				**<0.001**			
Grade 1/2	456 (49%)	1.0			1		
Grade 3	483 (51%)	1.9	1.4-2.4		1.2	0.9-1.6	0.2
**Lymph node status**				**<0.001**			
Negative	482 (51%)	1.0			1		
Positive	457 (49%)	2.5	1.9-3.2		2.9	1.5-2.6	**0.000**
**BCNS score (quartiles)**							**0.002**
Quartile 1	249 (27%)	1.0			1		
Quartile 2	242 (26%)	1.04	0.7-1.5	0.8	0.9	0.6-1.4	0.8
Quartile 3	228 (24%)	1.7	1.2-2.5	**0.005**	1.5	1.0-2.2	**0.05**
Quartile 4	220 (23%)	1.7	1.7-3.5	**<0.001**	1.9	1.2-2.8	**0.002**

B) METABRIC Validation cohort (n = 771)

		**Unadjusted**			**Adjusted**		
**Variable**	n (%)	HR	95.0 % CI	P-value	HR	95.0 % CI	P-value
**Tumor size**				**<0.001**			
≤ 20 mm	319 (41%)	1.0			1		
> 20 mm	452 (59%)	2.0	1.5-2.7		1.812	1.3-2.5	**<0.001**
**Histologic grade**				**<0.001**			
Grade 1/2	363 (47%)	1.0			1		
Grade 3	408 (53%)	1.7	1.2-2.2		1.118	0.8-1.6	0.5
**Lymph node status**							
Negative	390 (51%)	1.0		**<0.001**	1		
Positive	381 (49%)	2.9	2.1-3.8		2.403	1.8-3.2	**<0.001**
**BCNS score (quartiles)**							
Quartile 1	196 (25%)	1.0			1		
Quartile 2	186 (24%)	1.3	0.-1.9	0.3	1.276	0.8-2.0	0. 3
Quartile 3	169 (22%)	2.2	1.5-3.2	**<0.001**	2.003	1.3-3.1	**0.002**
Quartile 4	220 (29%)	2.0	1.4-3.0	**<0.001**	1.844	1.2-2.9	**0.008**

HR=Hazard ratio, CI=Confidence interval, n=number of patients. Missing cases, Validation cohort: Histologic grade, n=73; Tumor size, n=20; Follow-up time, n=2.

Supplementary Table S5.

Cox regression multivariate survival analysis (METABRIC Discovery cohort; Luminal subset only.
The Breast Cancer Necrosis Signature (BCNS) score was included as continuous variable. n=734.

		Unadjusted			Adjusted		
**Variable**	**n (%)**	**HR**	**95% CI**	**P-value**	**HR**	**95.0% CI**	**P-value**
**Tumor size**				**<0.001**			**<0.001**
≤ 20 mm	329 (45%)	1.0			1.0		
> 20 mm	405 (55%)	2.2	1.6-3.0		1.8	1.3-2.6	
**Histologic grade**				**<0.001**			0.05
Grade 1/2	435 (59%)	1.0			1.0		
Grade 3	299 (41%)	1.7	1.2-2.3		1.4	1.0-1.9	
**Lymph node status**							**<0.001**
Negative	400 (54%)	1.0		**<0.001**	1.0		
Positive	334 (46%)	2.2	1.6-3.0		1.8	1.3-2.5	
**BCNS score**	734 (100%)			**0.006**	1.009	1.002-1.016	**0.01**

HR=Hazard ratio, CI=Confidence interval, n=number of patients.

Supplementary Table S6.

Prediction of basal-like subtype by gene expression, multivariate logistic regression analyses.
A) TCGA; B) METABRIC discovery; C) METABRIC validation data (all microarray data).

Variables	n (%)	OR (95% CI)	P-value
**A) TCGA cohort, n = 505**			
**BCNS**	505 (100%)	1.082 (1.028-1.138)	**0.003**
**CK5**	505 (100%)	1.9 (0.6-5.5)	0.27
**P-cadherin**	505 (100%)	0.5 (0.15-1.92)	0.34
**EGFR**	505 (100%)	0.6 (0.19-1.65)	0.30
**B) METABRIC discovery cohort, n=939**			
**BCNS**	939 (100%)	1.082 (1.058-1.107)	**<0.001**
**CK5**	939 (100%)	2.7 (1.4-5.1)	**0.003**
**P-cadherin**	939 (100%)	1.061 (0.64-1.75)	0.82
**EGFR**	939 (100%)	0.42 (0.23-0.78)	**0.005**
**C) METABRIC validation cohort, n=845**			
**BCNS**	845 (100%)	1.051 (1.041-1.061)	**<0.001**
**CK5**	845 (100%)	3.2 (2.1-5.0)	**<0.001**
**P-cadherin**	845 (100%)	0.8 (0.6-1.1)	0.15
**EGFR**	845 (100%)	1.2 (0.8-1.8)	0.3

n=number of patients; OR=odds radio; CI=confidence interval; CK5, cytokeratin 5; EGFR, epidermal growth factor receptor. The basal-like subtype was compared with non-basal types (Luminal A; Luminal B; HER2-enriched subtypes). Normal breast-like cases were excluded. All variables were included in the analysis as continuous variables.

Supplementary Table S7.

Top 10 gene sets enriched in tumors with necrosis from the Molecular signatures Database (MSigDB). Categories: C2/Curated, C5/Gene ontology/biological processes, C6/Oncogenic signaling and Hallmarks. For C2 curated gene sets, the list is manually selected after interest with original ranking as indicated (all with FDR < 1.97).

GENE SET (MSigDB)	FDR (%)
**C2 CURATED (RANK)**	
FISCHER_G2_M_CELL_CYCLE (2)	2.0
WHITFIELD_CELL_CYCLE_G1_S (6)	1.5
BENPORATH_ES_1 (9)	1.3
MANALO_HYPOXIA_DN (11)	1.2
WONG_EMBRYONIC_STEM_CELL_CORE (13)	1.1
BENPORATH_ES_CORE_NINE_CORRELATED (16)	1.1
WHITFIELD_CELL_CYCLE_G2 (22)	1.6
SARRIO_EPITHELIAL_MESENCHYMAL_TRANSITION_UP (35)	1.8
BENPORATH_PROLIFERATION (36)	1.8
**C5 ONTOLOGY**	
GO_MEIOTIC_CHROMOSOME_SEGREGATION	5.0
GO_CHROMOSOME_ORGANIZATION_INVOLVED_IN_MEIOTIC_CELL_CYCLE	5.9
GO_REGULATION_OF_CHROMOSOME_SEGREGATION	6.1
GO_METAPHASE_ANAPHASE_TRANSITION_OF_CELL_CYCLE	6.1
GO_CHROMOSOME_SEPARATION	6.2
GO_CHROMOSOME_SEGREGATION	6.3
GO_NEGATIVE_REGULATION_OF_CHROMOSOME_SEGREGATION	6.6
GO_NUCLEAR_CHROMOSOME_SEGREGATION	6.7
GO_REGULATION_OF_CHROMOSOME_SEPARATION	7.0
GO_MITOTIC_NUCLEAR_DIVISION	7.0
**C6 ONCOGENIC**	
E2F1_UP.V1_UP	1.2
GCNP_SHH_UP_LATE.V1_UP	1.3
RPS14_DN.V1_DN	2.1
CSR_LATE_UP.V1_UP	3.7
PRC2_EZH2_UP.V1_DN	4.3
GCNP_SHH_UP_EARLY.V1_UP	5.6
VEGF_A_UP.V1_DN	5.6
SRC_UP.V1_DN	5.7
HOXA9_DN.V1_DN	5.8
CSR_EARLY_UP.V1_UP	6.0
**HALLMARKS**	
HALLMARK_G2M_CHECKPOINT	0.2
HALLMARK_E2F_TARGETS	0.7
HALLMARK_MYC_TARGETS_V1	0.7
HALLMARK_MTORC1_SIGNALING	2.4
HALLMARK_MITOTIC_SPINDLE	2.5
HALLMARK_MYC_TARGETS_V2	2.9
HALLMARK_SPERMATOGENESIS	7.0
HALLMARK_UNFOLDED_PROTEIN_RESPONSE	8.0

FDR= false discovery rate, MSigDB=Molecular signatures Database.
